# Effect of cocooning conditions on the structure, carbon and nitrogen isotope ratios of silks

**DOI:** 10.1371/journal.pone.0291769

**Published:** 2023-09-21

**Authors:** Hao Li, Yujie He, Liling Jia, Yong Liu, Dan Yang, Shuai Shao, Gang Lv, Hailiang Yang, Hailing Zheng, Xuhong Cui, Yang Zhou, Zhiqin Peng

**Affiliations:** 1 School of Materials Science and Engineering, Zhejiang Sci-Tech University, Hangzhou, China; 2 Institute of Textile Conservation, Zhejiang Sci-Tech University, Hangzhou, China; 3 China National Silk Museum, Hangzhou, China; 4 College of Life Science, China Jiliang University, Hangzhou, China; Universidad de la Republica Uruguay, URUGUAY

## Abstract

The stable isotope technique provides the possibility to trace ancient textiles because the technique is associated with advantages such as trace indication, fast detection, and accurate results. Since different cocooning conditions may impact cocoons even under identical habitats, it is important to investigate the effects of different cocooning temperatures and humidity on the isotope incorporation values in the cocoons. In this study, silk fibers were reeled under different conditions of temperature and humidity, followed by analysis of the secondary structure of cocoon proteins and isotope incorporation patterns. We found that the deviations in carbon isotope values of silk under different cocooning conditions could reach up to 0.76‰, while the deviation in carbon isotope values at different locations of a single silk was 2.75‰. Further, during the cocooning process, depletion of the ^13^C-isotope at different locations of the silk fibers was observed, reducing the δ^13^C values. We proposed that the changes in carbon isotopes in silk were related to the content of sericin and silk fibroin in silk. Finally, we did not observe a significant difference in isotope ratios in degummed cocoons. In summary, the ^13^C isotope was enriched in sericin, whereas ^15^N was enriched in fibroin, and these findings provide basic information for tracing the provenance of silks.

## 1. Introduction

Silkworm has played a crucial role and remained important for Chinese civilization. Silk is among the best-known textile materials and is available globally [1]. Huge amounts of archaeological fabric have been excavated along the Silk Road in recent decades, providing important historical information on ancient culture, economy, and technology [2]. Silk fabric is made up of protein and is therefore susceptible to degradation by several factors, such as light, humidity, heat, microorganisms, acid, and alkali. After unearthing, it is frequently impossible to completely preserve silk cultural relics. Further, due to the artistic and cultural values, accurately determining the origin of silk cultural relics is desirable, and historians and archaeologists are in search of an appropriate method, underscoring the importance of the study of silk.

Silk is produced by fully grown silkworms. However, silk synthesis is affected by various factors, such as temperature, humidity, and silkworm metabolism. Temperature and humidity are key ecological factors, and the effects of environmental temperature on species distribution are well known [3, 4]. Silkworm, which is found in many provinces of China, is also affected by changes in temperature and humidity. For example, the daily mean temperature in Sichuan Province is 20−23°C, with an average humidity of approximately 75.57%. On the other hand, the daily mean temperature and average humidity in most areas of Shandong Province are 23−29°C and above 80%, respectively. Hence, similar to other ectotherms, the fitness of silkworms depends highly on adaptations to different temperatures [5]. Furthermore, humidity also plays a crucial role in silk synthesis, particularly the silk composition. A single silk molecule glued with sericin protein consists of 70−80% fibroin protein, a few protease inhibitors, and other functional proteins [6]. The silk gland of the silkworm is the main processing site for these proteins and has three regions with different functions [7]. Changes in these regions of the silk gland result in changes to the silk. Further, changes in silk synthesis are accompanied by structural changes, primarily the conversion of α-helix and random coils to *β*-sheets; however, changes also involve protein crystallization. Previous studies have shown the existence of a certain correlation between the crystallization process and location of a cocoon [8], and the crystallization of the middle layer is relatively higher than that of the other layers of the cocoon.

Stable isotope ratio analysis is a relatively new technology used for food source analysis and textile traceability [9, 10]. Using hydrogen and oxygen stable isotope technology, the possibility of tracing the origin of the Turin Shroud (linen) was proposed [11]. Further, it was feasible to trace ancient wool fabric using light stable isotopes of carbon, nitrogen, and hydrogen, but the influence of climate and grazing conditions on the accuracy of data should be considered [12]. Stable isotopes of silk unearthed in Famen Temple (Shannxi Province, China) were determined by using isotope ratio mass spectrometry [13]. The authors pointed out that the origin of those silk fabrics was expected to be traced by combining relevant information of sericulture in Tang Dynasty. In addition, stable isotope technology is a highly efficient and accurate tool for tracing origin, and the technology can detect small differences in the isotopic composition of natural silk influenced by external and internal factors [14], allowing for the study of silk’s origin and changes.

In this study, attenuated total reflection-Fourier transform infrared (ATR-FTIR) spectroscopy and X-ray diffraction (XRD) were employed to analyze the secondary structure of silk proteins. Further, using the isotope technique, changes in the carbon and nitrogen isotopes of silk were evaluated. We also investigated the effects of different cocooning conditions and cocooning processes on silk and elucidated the isotope changes in silk between untreated and degummed cocoons.

## 2. Materials and methods

### 2.1 Rearing of silkworms

Larvae of the Qiufeng × Baiyu *Bombyx mori* strain were provided by Zhejiang Academy of Agricultural Sciences and reared on fresh mulberry leaves at 25°C with 75% relative humidity (RH) under standard conditions until their fifth instar [15]. The fifth instar larvae were randomly divided into the following nine groups (n = 50*9) to finish cocooning: (i) HH (short for high temperature and high humidity, 30.0 ± 2°C, 90% ± 10% RH), (ii) HN (short for high temperature and normal humidity, 30.0 ± 2°C, 70% ± 10% RH), (iii) HL (short for high temperature and low humidity, 30.0 ± 2°C, 50% ± 10% RH), (iv) NH (short for normal temperature and high humidity, 25.0 ± 2°C, 90% ± 10% RH), (v) NN (short for normal temperature and normal humidity, 25.0 ± 2°C, 70% ± 10% RH), (vi) NL (short for normal temperature and low humidity, 25.0 ± 2°C, 50% ± 10% RH), (vii) LH (short for low temperature and high humidity, 21.0 ± 2°C, 95% ± 5% RH), (viii) LN (short for low temperature and normal humidity, 21.0 ± 2°C, 85% ± 5% RH), and (ix) LL group (short for low temperature and low humidity, 21.0 ± 2°C, 75% ± 5% RH). Each experiment was performed in triplicate (S1 Table in S1 File). Further, larvae were kept under a 12 h light and 12 h dark condition.

### 2.2 Preparation of silk

After peeling the husks from the cocoons of the nine groups (reared under nine different cocooning conditions), the cocoons were placed in deionized water at 60°C for 15 min at a bath ratio of 1:100 and subsequently reeled using a manual grain reeling machine at a speed 100 times/min. For each sample (except for the NH group), reeling was required for approximately 1000 times (0.2 m × 1000 turns) to reach to the end of the silk fiber with evenly divided five sections, leaving a thin palette. In the NH group, the silk fibers could not be reeled smoothly and hence only four sections were collected, with thicker palettes. For the isotopic analysis, all the silk samples were washed five times using deionized water and 75% ethanol mixture solution, dried at 60°C for 2 h, and stored in a desiccator.

### 2.3 Degumming treatment

Cocoons were incubated in a 0.5% (w/w) Na_2_CO_3_ aqueous solution at 98°C ± 2°C for 1 h at a bath ratio of 1:100, and this process was repeated four times. Subsequently, the degummed cocoon layers were soaked in 1% (w/w) hydrochloric acid at 25°C for 1 h, washed using deionized water, and dried at 60°C.

### 2.4 Optical microscopy testing

The diameter of silk fibers was determined using an optical microscope (PH100-3A41L-EP, Phenix Optical Company Limited, China). Seven cocoons were evaluated for each group and tested five times for each section.

### 2.5 Fourier transform infrared spectroscopy analysis

All the specimens were carried out using a Thermo Scientific Nicolet is50 FT-IR spectrometer for the structure analysis with the resolution of 4 cm^-1^, spectral range of 400-4000cm^-1^, and 32 scans [16]. For secondary structure analyses, each of the fiber spectra underwent the following process: 1) Smoothing with an SG quad-cubic function of 7–9 points, taking care not to alter any diagnostic feature of the spectra; 2) Spectrum truncation down to the 1300–1180 cm^-1^ range (Amide Ⅲ region); 3) Baseline correction using a liner function connecting the two extremes of the truncated spectra; 4) Each spectrum was normalized to the maximum absorbance value of the Amide Ⅲ band. Operations 1–4 were carried out using the Agilent Resolution Pro software (Agilent technologies). Each resultant spectrum was deconvoluted and fitted using the multipeak fitting package of the Igor Pro software, version 7 (WaveMetrics, Inc). First, the second derivative of the convoluted spectra was used to locate the position of bands. Then, the spectra were deconvoluted using Gaussian curves and a constant baseline (constrained at zero absorbance), in two steps: The position and width of the bands were hold; the height of all bands constrained to a maximum of 80 and a minimum of 0; the fitting was then iterated until no changes were reported between two successive iterations; 2) The width was let change in the 0–20 limit, and the height was let change with a minimum limit of 0; the fitting was iterated until no changes were reported between two successive iterations. The resultant deconvoluted bands composing amide Ⅲ were assigned to the different secondary structures of the protein.

### 2.6 X-ray diffraction analysis

X-ray diffraction analysis (XRD, D8 discover, Bruker AXS LTD, Germany) was used to identify the crystalline phase of the samples. X-ray scanning was performed at diffraction angles from 5°−50° (2θ) with a scan rate of 2°/min.

### 2.7 δ^13^C and δ^15^N isotopic analysis

The silk samples were cut into small powders using surgical scissors. Approximately 0.2−0.5 mg of each silk sample was placed in tin capsules and introduced sequentially into an elemental analyzer (Flash 2000HT, Thermo Finnigan, USA) coupled to an isotope-ratio mass spectrometer (MAT253, Thermo Finnigan, USA). The isotopic values are reported in the ‘δ’ notation in ‘‰’ relative to the international standards Vienna Pee Dee Belemnite (VPDB). The ‘δ’ values were calculated as follows:

$$\delta =(\frac{R_{samp}}{R_{std}}-1)\times 1000$$

where R_std_ is either the ^13^C/^12^C ratio of the VPDB for carbon or the ^15^N/^14^N ratio of atmospheric N_2_, and R_samp_ is the ratio for silk samples [17]. The ratios of the stable isotopes were adjusted against the following international standard references: IAEA-CH-7 (polyethylene) for the δ^13^C value and IAEA-600 (caffeine) for the δ^15^N value. The analytical precision was lower than ± 0.2‰ for both C and N.

## 3. Results

### 3.1 Comparison of silk diameter

To study the effect of different cocooning conditions on the silk performance index, we analyzed the silk diameter, and the average diameter of five sections obtained from high temperature groups were 7.81 μm, 8.37 μm, 8.17 μm, 7.81 μm, and 7.50 μm, whereas the average diameter of five sections obtained from normal and low temperature groups were 7.77 μm, 8.63 μm, 8.48 μm, 7.85 μm, and 7.98 μm and 8.35 μm, 8.81 μm, 9.00 μm, 9.00 μm, and 8.98 μm, respectively (S2 Table in S1 File). In all three types of groups, the silk diameter first increased and then decreased. This result is consistent with [18]. Further, the diameter of the silk from low temperature groups was larger than that from high temperature groups.

### 3.2 Comparison of secondary structure of silk

**Fig 1:**
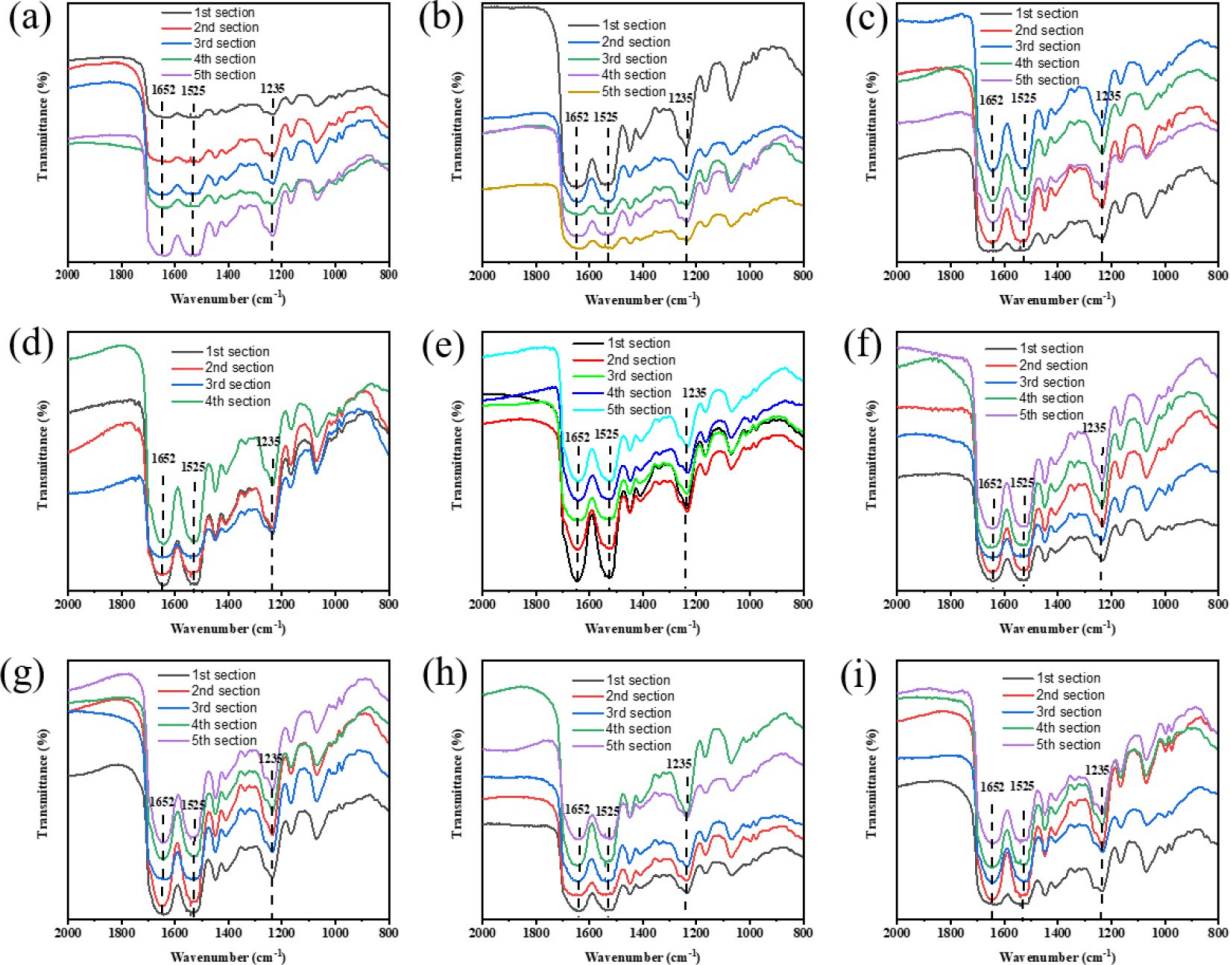
FTIR spectrum of silk reeled under different cocooning conditions. The first, second, third, fourth and fifth sections represent 0−200 m, 200−400 m, 400−600 m, 600−800 m, and 800−1000 m of the silk, respectively. Each silk section represents a different location in the silk. (a: HH; b: HN; c: HL; d: NH; e: NN; f: NL; g: LH; h: LN; i: LL).

**Fig 2:**
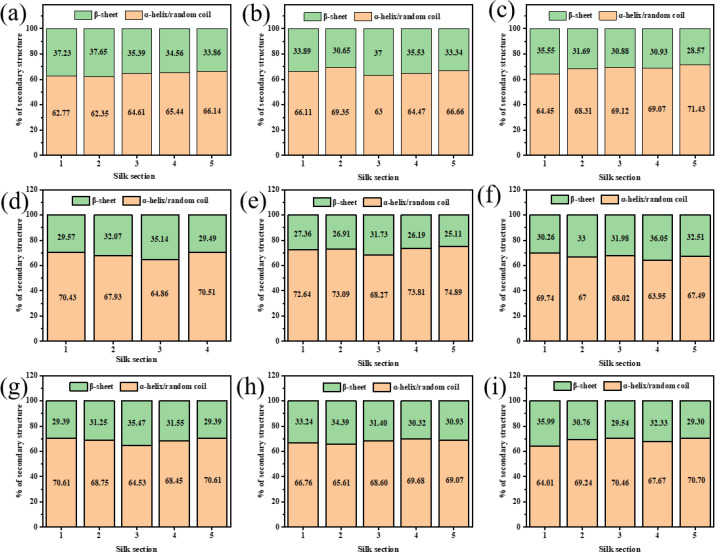
Content of different secondary structures of silk reeled under different cocooning conditions. The first, second, third, fourth, and fifth sections represent 0−200 m, 200−400 m, 400−600 m, 600−800 m, and 800−1000 m of the silk, respectively. Each silk section represents a different location in the silk. (a: HH; b: HN; c: HL; d: NH; e: NN; f: NL; g: LH; h: LN; i: LL).

To study the effect of different cocooning conditions on silk structure, ATR spectroscopy was used to identify functional groups. Fig 1 shows the characteristic peaks of silk samples. The absorption peaks at 1647 cm^-1^ and 1233 cm^-1^ were attributed to random coils, *α*-helices, or both, whereas the peaks at 1516 cm^-1^ and 1270 cm^-1^ represented *β*-sheet structure, reflecting crystallinity of cocoon proteins [19, 20]. Although the position and shape of characteristic peaks in all the groups were similar, the intensity of peaks differed among groups, especially for the NH group (Fig 1D) that lacked the absorption peaks at 1647 cm^-1^ and 1516 cm^-1^. In LL and NL groups (Fig 1F and 1I), a small peak at 1745 cm^-1^ appeared, representing the existence of aldehyde or ketonic groups [21]. Amide III was used to analyze secondary structures, and the contents of *β*-sheets in each section of silk were calculated (Fig 2). We found that the *β*-sheet content increased as the silk section increased (from 1 to 5) in the high temperature groups (Fig 1A–1C). In the normal and low temperature groups (Fig 1D–1F), the content of *β*-sheets in third or fourth section was higher than those in other sections in most cases.

### 3.3 Comparison of crystal structure of silk

**Fig 3:**
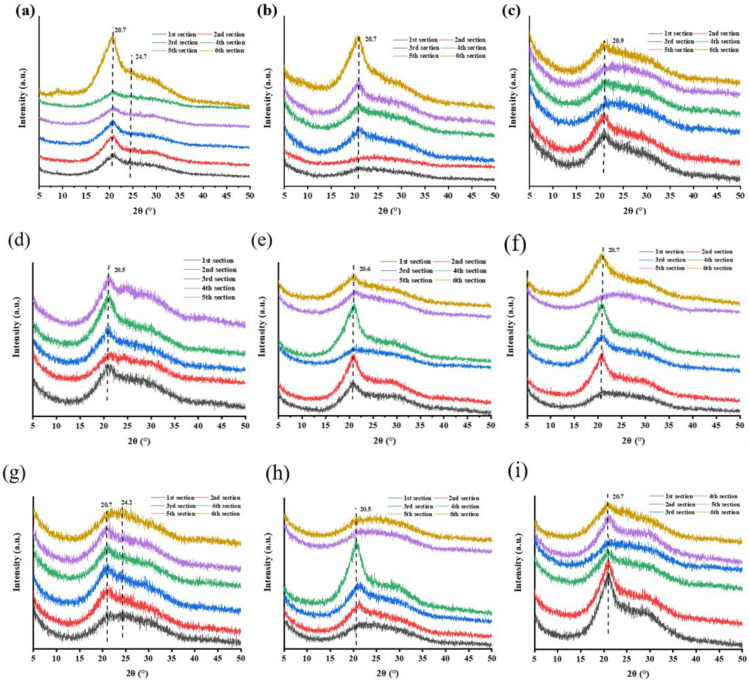
XRD analysis of silk fibers reeled under different cocooning conditions. The first, second, third, fourth, fifth, and sixth sections represent 0−200 m, 200−400 m, 400−600 m, 600−800 m, 800−1000 m of the silk and the pelettes, respectively. Each silk section represents a different location in the silk. (a: HH; b: HN; c: HL; d: NH; e: NN; f: NL; g: LH; h: LN; i: LL).

XRD was used to further characterize the secondary structures of silk. A diffraction peak at approximately 20.7° was observed, which was attributed to the *β*-sheet structure [22]. Further, the intensity of the diffraction peak in the middle part of silk (third or fourth silk section) was higher than those from other sections in most groups (Fig 3). In the high temperature groups (Fig 3A–3C) and the NL group (Fig 3F), the peaks turned into strong abruptly for the sixth section. For the LL (Fig 3I) and HL (Fig 3C) groups, the peaks in the initial sections (the first and second silk sections) were higher than those in the later sections. It indicates that the crystallinity of silk fibers in a single cocoon change with the change of temperature and humidity. Although the pelettes (the innermost layer of cocoon that naturally falls during reeling [23], named the sixth section of cocoon herein) is also protein, it is lamellar rather than fibrous. Therefore, the sixth section is different from the other sections in terms of crystallinity. These results indicate that the changes of secondary structure of silk protein are related to the physiological and metabolic conditions of silkworms. Besides, the intensity of the diffraction peak in the middle part of silk (third or fourth silk section) is higher than those from other sections in most groups, except the LL and HL groups. These results indicate that high humidity is conducive to the crystallization of silk protein to some extent.

### 3.4 Distribution of carbon and nitrogen isotope values of silk reeled under different cocooning conditions

**Fig 4:**
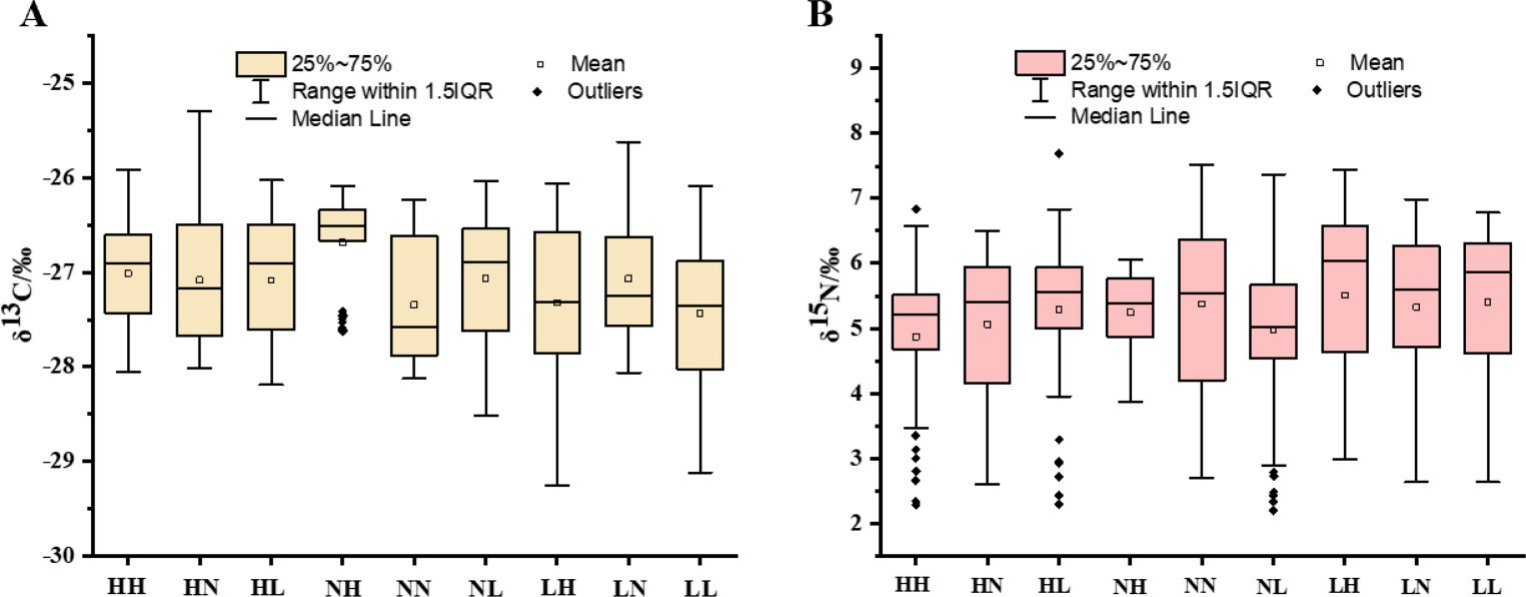
Replicate measurements of δ^13^C (A) and δ^15^N (B) values of silk samples reeled under different cocooning conditions. Box plots show the distribution of all measurements under each condition. For each silk sample, three measurements were made.

The δ^13^C and δ^15^N values of silk samples ranged from (mean ± SE) -27.44 ± 0.76‰ to -26.68 ± 0.49‰ (P < 0.05) and 4.87 ± 1.04‰ to 5.51 ± 1.26‰ (P < 0.05), respectively. Further, significant differences were observed in the distribution of δ^13^C values of silk samples from the NH group when compared to those from other groups. The distribution of δ^13^C values in silk samples from high (HH, HN, and HL) and low (LH, LN, and LL) temperature groups did not differ much. However, the distribution of δ^13^C values of silk samples from the NH group showed a significant difference in comparison to normal temperature groups (Fig 4A); however, the δ^13^C values of silk samples from the NH group had many outliers. The NH group showed significantly higher δ^13^C values (0.76‰) than that of the LL group (S3 Table in S1 File). On the other hand, the δ^15^N values of the silk samples in all the groups showed little variation, with a maximum deviation of 0.64‰ (S4 Table in S1 File). HH, HL, NH, and NL groups had a narrower distribution of δ^15^N values than the other five groups. Further, the δ^15^N values of silk samples from the HH, HL, and NL groups had many outliers (Fig 4B). The δ^13^C and δ^15^N values of the average of six sections of silk were close to the δ^13^C and δ^15^N values between the third and fourth sections of silk samples (S3 and S4 Tables in S1 File).

### 3.5 Analysis of carbon isotope values at different locations of silk reeled under different cocooning conditions

**Fig 5:**
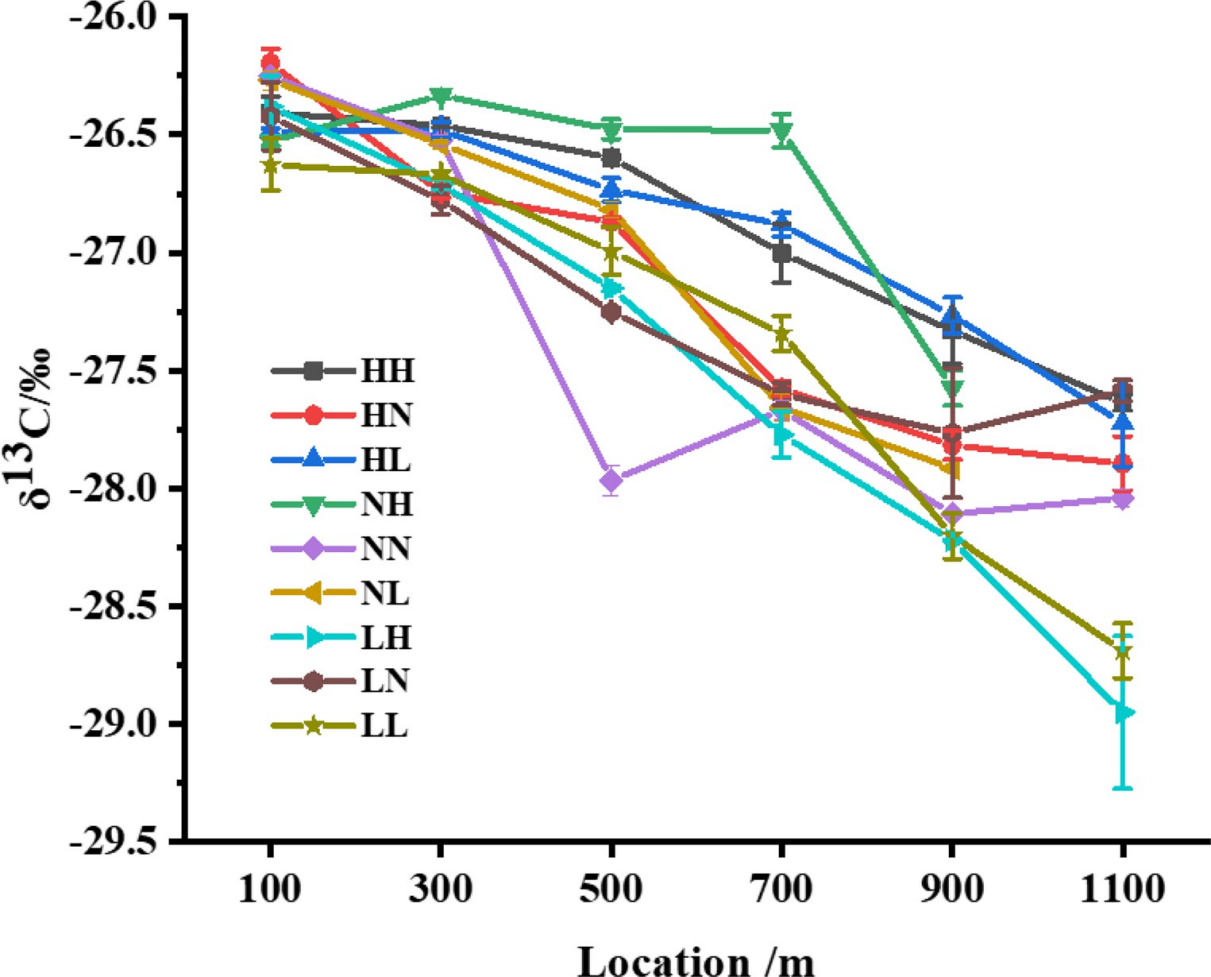
Changes in δ^13^C values at different locations in silk reeled under different cocooning conditions.

The δ^13^C values at different locations of silk reeled under different cocooning conditions ranged from (mean ± SE) -26.20 ± 0.06‰ to -28.95 ± 0.33‰ (P < 0.05), with an overall decline of approximately 2.56‰ in the LH group (Fig 5). Overall, the changes in δ^13^C values in all the nine groups were identical, with no difference at the beginning and a slight difference toward the end of the silk fiber. The absolute difference between the highest and lowest isotope values was 1.49‰ at the same location.

## 3.6 Analysis of carbon and nitrogen isotope values of silk sections

**Fig 6:**
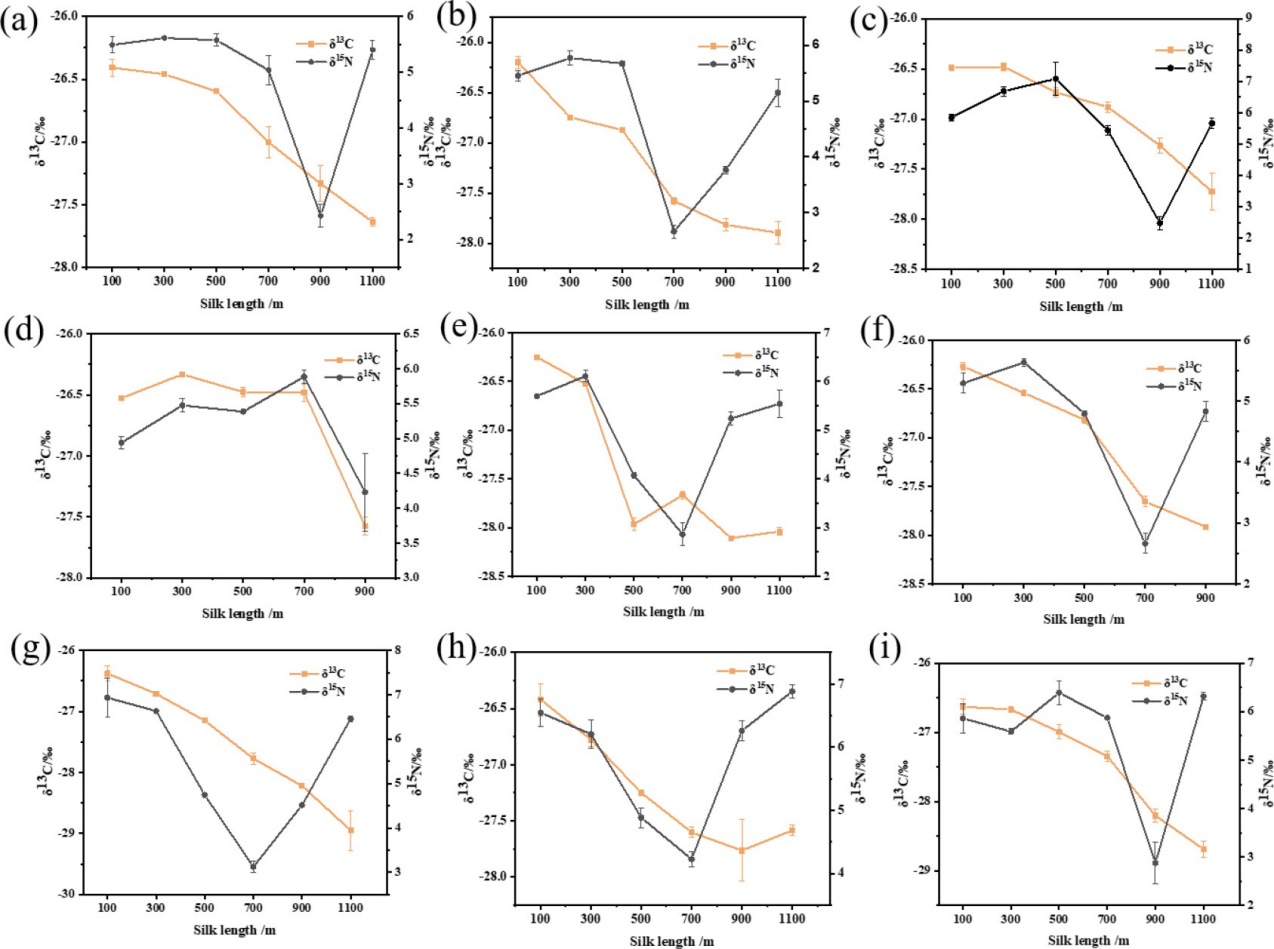
Changes of δ^13^C and δ^15^N values at different locations of silk reeled under different conditions. (a: HH; b: HN; c: HL; d: NH; e: NN; f: NL; g: LH; h: LN; i: LL).

The δ^13^C values of the first and palette sections of the silk ranged from (mean ± SE) -26.63 ± 0.11‰ to -26.20 ± 0.06‰ (P < 0.05) and -28.95 ± 0.33‰ to -27.59 ± 0.05‰ (P < 0.05), respectively. The δ^13^C values showed a decreasing trend along the different locations of the silk fiber in the nine different groups. The δ^15^N values also showed a similar pattern, with a decrease until the middle and late stages of the cocooning processes, except for the NH group (Fig 6D), which showed the opposite tendency. Further, the δ^15^N values showed a maximum deviation of approximately 3‰ during the middle and late stages of the cocooning process, but the difference between the start and finish δ^15^N values was negligible (Fig 6).

### 3.7 Analysis of carbon and nitrogen isotope values in degummed cocoons

**Fig 7:**
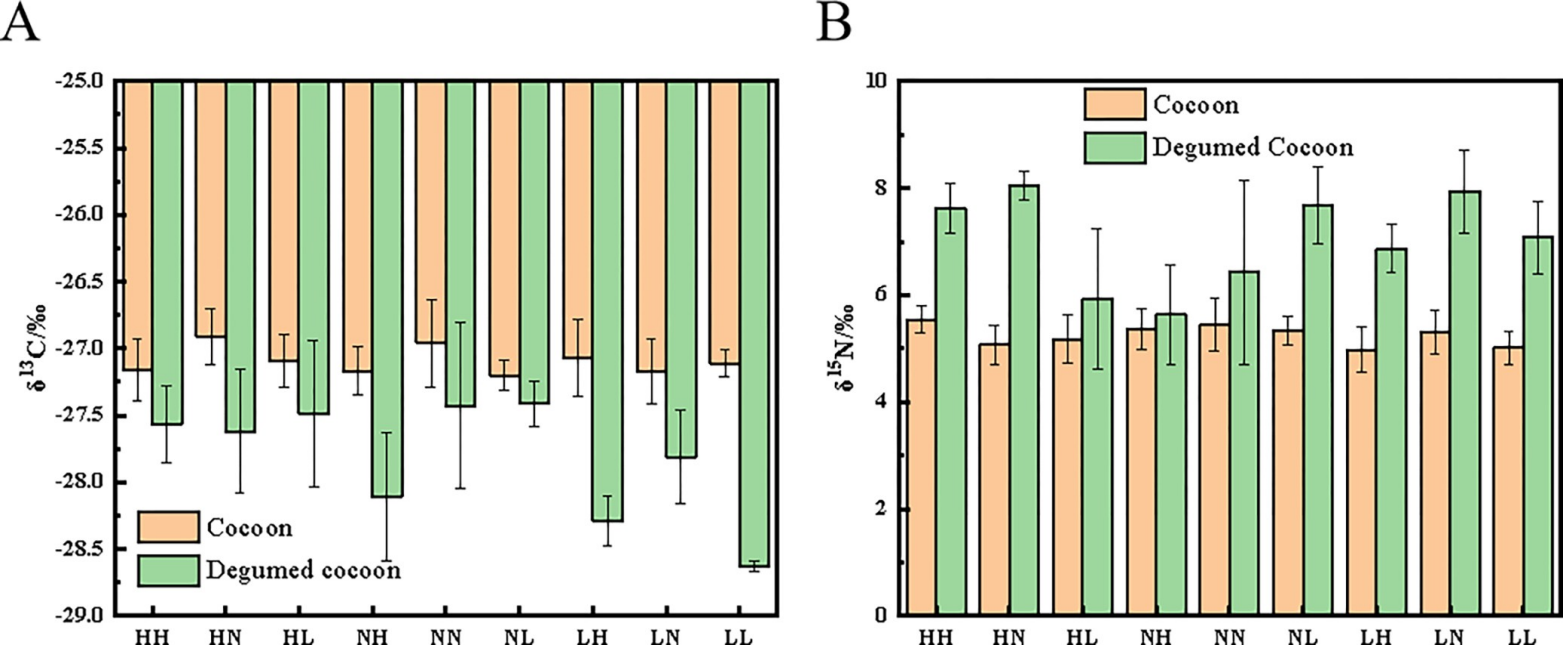
δ^13^C (A) and δ^15^N (B) values in cocoons and degummed cocoons. Descriptive statistics in the figures are provided as the mean ± SD.

Degummed cocoons showed a trend of δ^13^C and δ^15^N values similar to that of cocoons under the nine different cocooning conditions. However, degummed cocoons had lower δ^13^C values in comparison to the raw cocoons (Fig 7A). The overall δ^13^C values of raw and degummed cocoons in the nine different groups ranged from -27.20 to -26.91‰ and -28.63 to -27.41‰, respectively, while the overall δ^13^C values ranged between 0.21 and 1.52‰ (S5 Table in S1 File). In contrast, degummed cocoons showed higher δ^15^N values than those of raw cocoons. The overall δ^15^N values of raw and degummed cocoons ranged between 4.98 and 5.54‰ and 5.63 and 8.04‰, respectively, with overall δ^15^N values ranging from 0.27 to 2.96‰ (S5 Table in S1 File). The lowest δ^13^C (0.21‰) and δ^15^N (0.27‰) values were recorded in the NL and NH groups, respectively, whereas the highest δ^13^C (1.52‰) and δ^15^N (0.27‰) values were observed in the LL and HN groups, respectively.

## 4. Discussion

In this study, we measured the diameters of silk samples reeled under different cocooning conditions and locations (S2 Table in S1 File). Variations in silk diameter may be related to silking behavior. The formation of natural silk is a dry spinning process, where the fibrosis of silk protein is achieved by shearing action in the gland cavity and stretching action of the head during silking [24]. During the whole cocooning process, the swing rate of the head of the silkworm first increases and then decreases, causing an increment in the drafting effect followed by a fading of the effect. Moreover, the shear effect in the gland cavity also changes during the cocooning process. These factsexplain our results of an increased diameter of silk followed by a decrease during the cocooning process (S2 Table in S1 File).

FTIR and XRD results revealed that the crystallinity trend was consistent with the β-sheet content. Consistent with previous findings [25], the crystallinity of the middle part of a cocoon (the third or fourth silk section) was higher in comparison to other parts of the cocoon in most cases. Due to the unsuitable temperature and humidity and the continuous consumption of energy during the silking process of the silkworm, the crystallinity of the first and second sections of silk fibers in the HL and LL groups was higher than that of the other silk fibers. Meanwhile, the silking speed of the silkworm increased with the increasing cocooning temperature. The high temperature was conducive to the movement of peptide chain segments in proteins and the formation of regular stacked silk structures [26]. At the same time, cocooning humidity affected the dispersion of water from the silk, thus affecting the microstructure of the silk. The humidity in the NH group was high enough to cause condensation, affecting the silking behavior of silkworms and eliminating the absorption spectra of a few functional groups in the fifth section of the silk fiber [27].

When we measured the isotope ratio values of silk samples reeled under different temperature and humidity conditions, the carbon and nitrogen isotope values of the silk (Fig 6) varied slightly under each cocooning condition, suggesting that the thermal and moist stresses have a certain effect on the silking behavior of a silkworm and the isotope changes in silk. It is known that environmental temperature is one of the most significant factors affecting the lives of organisms, and organisms are essentially required to tolerate varying degrees of thermal stress [28]. Silkworms display a faster speed of cocooning at high temperatures. Our isotope value data also supported this theory, further concurring with the fact that silkworms show great adaptability to survive multiple environmental stresses [5].

The silkworm’s cocooning process is also related to its own physiological conditions [29]. Many studies have shown that silk is mostly composed of fibroin and sericin, which are synthesized under the synergistic action of three distinct regions of silk glands [30, 31]. The posterior and middle regions synthesize silk fibroin and sericin, respectively. In contrast, the anterior region, which is a narrow duct, plays an important role in the process of spinning the silk proteins [7, 32]. Complete silk is continuous, with lengths of 700−1500 m reeled from its cocoon [33]. Previous studies have also reported the properties of silk by dividing it into five parts [34]. In the present study, we found that the carbon and nitrogen isotope values of silk had similar changes under different environmental conditions, and the heavy elements of carbon were depleted, causing a decrease in the carbon isotope ratio during the silking process. Our results (Fig 6, S3 Table in S1 File) also proved that within the limited silk length, carbon isotope values varied, reaching up to 2.75‰ from the beginning to the end of the cocooning process. Previous studies have reported that isotope technology is applied to human hair detection and can reflect on the human diet at a certain time [35]. The main component of human hair is keratin, which is the product of human metabolism [36, 37]. Since silk is also made up of proteins synthesized by the silkworms, the changes in isotope ratio values might also reflect the diet of silkworms; further, the transformation metabolism between mulberry leaf protein and silk protein can also be revealed. Evidence suggests that the sequence of mulberry leaves consumption by silkworms corresponds to the time of silk protein transformation [38]. Furthermore, the presence of silk protein in a silkworm could be one of the causes of isotope changes in silk. Carbon and nitrogen are the basic elements of fibroin and sericin in silk. Previous studies have found that the contents of fibroin and sericin in the silks released by a silkworm at different cocooning processes are also different [34]. For instance, for silk reeled from a cocoon divided into five layers, the contents of sericin are 32.42%, 23.15%, 19.79%, 17.86%, and 17.78% from the outer layer to the inner layer [34]. These results (Fig 7, S5 Table in S1 File) are in accordance with our data on carbon isotope ratio values in silk. It was also presumed that sericin in silk may contain more heavy carbon elements, whereas fibroin may contain more light carbon elements. Hence, with the decrease in the sericin content of silk, the carbon element content also decreases, causing a decline in the isotope ratio values in silk. These predictions are confirmed from our degumming experiments, where degummed cocoons had lower carbon isotope ratio values and higher nitrogen isotope ratio values than untreated cocoons.

A silkworm ingests protein from the mulberry leaves before the fifth instar, mainly for growth, and while the growth of a silkworm is completed after the fifth instar, it continues to eat mulberry leaves to store energy for subsequent stages [38]. Silk released from the silkworm is mostly converted from mulberry leaves at the fifth instar, and the remaining silk is converted from protein consumed by silkworms. In our isotope analysis, a turning point in δ^15^N values (Fig 6) was observed in the middle and late stages of the cocooning process. Previous studies have shown that the silk released at the late stages of the cocooning process was synthesized by the decomposition of its own protein [38]. However, silkworms mainly synthesize sericin, which is enriched in ^15^N in the early fifth instar, while synthesizing fibroin, which is enriched in ^14^N in the late fifth instar (Fig 6). These facts might explain the observed turning point of δ^15^N values and subsequent changes in silk fibers. However, more studies are required to prove such a relationship.

Notably, we noticed a certain relationship between the silk properties, structures, and isotopes. In the middle stage of the cocooning process, diameter and crystallinity of silk reached the maximum levels (S2 Table in S1 File, Fig 3). At the same time, the carbon and nitrogen isotope ratio values (S3, S4 Tables in S1 File) of the middle section of a cocoon could best represent its isotope ratio values for origin tracing testing.

Finally, we also found that the metabolism of silkworms had a greater influence on carbon isotope ratio values than that of cocooning conditions, indicating less attention needs to be paid to the influence of cocooning conditions at different locations when we try to trace the provenance of silks. The isotope ratio analysis is an effective method in the field of geographic origin detection. It has been successfully applied to rice [39], sea bass [40], red bell peppers [14], milk [41, 42], and wine [43]. In this study, we have provided the basic data to trace silk origin using the isotope technology.

In conclusion, this study showed that there were changes in the content of secondary structure in silk fibers during different cocooning processes. We show that the crystallinity of the silk reached its maximum at the middle stage of the cocooning process. Further, the isotope ratio values calculated in this study varied greatly during the whole cocooning process. The study also confirms that the influence of metabolic processes on silkworms was greater than that of cocooning conditions in terms of changes in carbon and nitrogen isotope values in silk. We further found that ^13^C was enriched in sericin, while ^15^N was enriched in fibroin. These results will help provide a scientific basis for the traceability of silk fabric’s origin. Future studies focusing on the isotope relationship between mulberry leaves and silk are needed to provide evidence of isotopic variations in silk.

## Supporting information

S1 File (DOCX)
