# Supplementary material for: Effect of cocooning conditions on the structure, carbon and nitrogen isotope ratios of silks
Source: PLoS One. 2023 Sep 21;18(9):e0291769. doi: 10.1371/journal.pone.0291769 (PMC10513321; doi:10.1371/journal.pone.0291769)
Supplement: S1 File — (DOCX) [file pone.0291769.s001.docx]

**Table S1** Statistic for temperature and humidity used in this study.

| Group | Temperature/℃ | Humidity/% |
| --- | --- | --- |
| HH | 29.03±1.12 | 88.16±3.50 |
| HN | 31.46±0.70 | 65.26±5.42 |
| HL | 31.63±0.62 | 55.95±5.76 |
| NH | 23.97±0.64 | 96.84±6.40 |
| NN | 26.37±0.90 | 66.21±5.81 |
| NL | 26.39±0.82 | 57.68±3.67 |
| LH | 19.10±1.81 | 91.73±6.80 |
| LN | 19.85±1.44 | 82.12±5.15 |
| LL | 20.12±1.73 | 75.91±6.41 |

**Table S2** Statistic for diameter of each silk segment from different temperature and humidity conditions

| Temperature  group | Humidity  group | diameter /μm | | | | | |
| --- | --- | --- | --- | --- | --- | --- | --- |
|  |  | 1st section（0~200m） | 2nd section（200~400m） | 3rd section（400~600m） | 4th section（600~800m） | 5th section（800~1000m） | AVG |
| H | H | 7.50±0.01 | 7.44±0.01 | 7.94±0.01 | 7.89±0.01 | 6.92±0.01 | 7.54±0.01 |
|  | N | 7.33±0.01 | 8.06±0.01 | 7.89±0.01 | 7.67±0.01 | 7.75±0.01 | 7.74±0.01 |
|  | L | 8.61±0.01 | 9.61±0.01 | 8.67±0.01 | 7.89±0.01 | 7.83±0.01 | 8.52±0.01 |
|  | AVG | 7.81±0.01 | 8.37±0.01 | 8.17±0.01 | 7.81±0.01 | 7.50±0.01 | — |
| N | H | 8.50±0.01 | 8.63±0.01 | 8.48±0.01 | 7.85±0.01 | — | 8.31±0.01 |
|  | N | 7.06±0.01 | 8.56±0.01 | 8.81±0.01 | 7.44±0.01 | 6.75±0.01 | 7.73±0.01 |
|  | L | 7.75±0.01 | 9.06±0.01 | 8.13±0.01 | 8.13±0.01 | 9.20±0.01 | 8.45±0.01 |
|  | AVG | 7.77±0.01 | 8.63±0.01 | 8.48±0.01 | 7.85±0.01 | 7.98±0.01 |  |
| L | H | 8.50±0.01 | 9.27±0.01 | 9.50±0.01 | 10.00±0.01 | 8.83±0.01 | 9.22±0.01 |
|  | N | 7.44±0.01 | 8.72±0.01 | 9.17±0.01 | 8.50±0.01 | 8.39±0.01 | 8.44±0.01 |
|  | L | 9.11±0.01 | 8.44±0.01 | 8.33±0.01 | 8.50±0.01 | 9.72±0.01 | 8.82±0.01 |
|  | AVG | 8.35±0.01 | 8.81±0.01 | 9.00±0.01 | 9.00±0.01 | 8.98±0.01 | — |
| All average | | 7.98±0.01 | 8.60±0.01 | 8.55±0.01 | 8.22±0.01 | 8.15±0.01 | — |

| Temperature  group | Humidity  group | δ^13^C /‰ | | | | | | |
| --- | --- | --- | --- | --- | --- | --- | --- | --- |
|  |  | 1st section（0~200m） | 2nd section（200~400m） | 3rd section（400~600m） | 4th section（600~800m） | 5th section（800~1000m） | 6th section（1000~1200m） | AVG |
| H | H | -26.35±0.36 | -26.69±0.22 | -26.75±0.12 | -27.19±0.20 | -27.37±0.10 | -27.57±0.36 | -27.01±0.55 |
|  | N | -26.25±0.07 | -26.50±0.24 | -26.69±0.61 | -27.36±0.41 | -27.67±0.15 | -27.75±0.15 | -27.08±0.66 |
|  | L | -26.35±0.15 | -26.29±0.17 | -26.77±0.10 | -27.34±0.34 | -27.83±0.43 | -27.70±0.12 | -27.09±0.66 |
|  | AVG | -26.31±0.22 | -26.54±0.36 | -26.90±0.29 | -27.43±0.36 | -27.61±0.33 | -27.72±0.18 | —— |
| N | H | -26.53±0.02 | -26.24±0.10 | -26.40±0.08 | -26.59±0.09 | -27.52±0.08 | -27.68±0.03 | -26.83±0.61 |
|  | N | -26.33±0.09 | -26.59±0.07 | -27.55±0.34 | -27.68±0.09 | -28.02±0.13 | -27.80±0.25 | -27.35±0.66 |
|  | L | -26.22±0.02 | -26.34±0.26 | -26.70±0.23 | -27.07±0.49 | -27.52±0.48 | -28.03±0.38 | -27.06±0.72 |
|  | AVG | -26.27±0.14 | -26.50±0.13 | -26.99±0.48 | -27.39±0.48 | -28.01±0.11 | -27.74±0.36 | —— |
| L | H | -26.31±0.18 | -26.69±0.02 | -27.21±0.22 | -27.81±0.07 | -27.56±0.88 | -28.31±0.61 | -27.32±0.83 |
|  | N | -26.11±0.28 | -26.56±0.22 | -27.00±0.25 | -27.40±0.19 | -27.83±0.20 | -27.51±0.14 | -27.07±0.63 |
|  | L | -26.58±0.38 | -26.80±0.33 | -27.08±0.23 | -27.34±0.07 | -27.91±0.23 | -28.56±0.37 | -27.44±0.76 |
|  | AVG | -26.33±0.34 | -26.72±0.26 | -27.03±0.33 | -27.48±0.23 | -27.94±0.22 | -28.12±0.61 | —— |
| All average | | -26.31±0.25 | -26.59±0.28 | -26.97±0.37 | -27.43±0.36 | -27.85±0.30 | -27.86±0.46 | —— |

**Table S3** Statistic for carbon isotope ratio values of each silk segment from different temperature and humidity conditions.

**Table S4** Statistic for nitrogen isotope ratio values of each silk segment from different temperature and humidity conditions.

| Temperature  group | Humidity  group | δ^15^N /‰ | | | | | | |
| --- | --- | --- | --- | --- | --- | --- | --- | --- |
|  |  | 1st section（0~200m） | 2nd section（200~400m） | 3rd section（400~600m） | 4th section（600~800m） | 5th section（800~1000m） | 6th section（1000~1200m） | AVG |
| H | H | 5.49±0.21 | 5.69±0.70 | 5.47±0.25 | 4.01±0.84 | 3.67±1.37 | 5.01±0.82 | 4.87±1.04 |
|  | N | 5.71±0.29 | 5.90±0.40 | 5.57±0.36 | 3.75±0.85 | 4.56±1.09 | 5.08±0.08 | 5.06±1.02 |
|  | L | 5.84±0.11 | 6.18±0.40 | 6.02±0.94 | 4.18±1.05 | 4.26±1.36 | 5.45±0.31 | 5.29±1.16 |
|  | AVG | 5.68±0.27 | 5.86±0.59 | 5.41±0.87 | 4.07±1.00 | 4.07±1.36 | 5.30±0.52 | —— |
| N | H | 5.94±0.09 | 5.74±0.25 | 5.67±0.24 | 5.37±0.46 | 4.38±0.31 | 5.68±0.19 | 5.25±0.62 |
|  | N | 6.24±0.64 | 6.58±0.53 | 3.97±0.32 | 3.52±0.81 | 5.81±0.86 | 6.07±0.78 | 5.37±1.36 |
|  | L | 4.01±0.02 | 5.72±0.85 | 6.04±0.81 | 4.72±1.61 | 3.62±1.71 | 4.87±0.47 | 4.97±1.39 |
|  | AVG | 5.81±0.74 | 6.05±0.73 | 4.91±0.90 | 4.00±1.39 | 4.94±1.74 | 5.15±0.91 | —— |
| L | H | 6.71±0.34 | 6.42±0.23 | 4.24±0.85 | 3.78±0.72 | 5.77±1.00 | 6.71±0.34 | 5.51±1.26 |
|  | N | 6.07±0.49 | 6.03±0.26 | 4.58±0.88 | 3.64±0.68 | 5.32±0.72 | 6.07±0.49 | 5.37±1.13 |
|  | L | 6.19±0.34 | 6.24±0.52 | 5.37±1.15 | 4.38±1.16 | 4.25±1.08 | 6.19±0.34 | 5.41±1.17 |
|  | AVG | 6.32±0.48 | 6.06±0.57 | 4.94±1.24 | 3.95±0.93 | 4.91±1.00 | 6.16±0.57 | —— |
| All average | |  | 5.99±0.63 | 5.09±1.03 | 4.01±1.09 | 4.64±1.35 | 5.53±0.82 | —— |

**Table S5** Carbon and nitrogen isotope ratio values of cocoons and degummed cocoons from different temperature and humidity conditions.

| Groups |  | Isotope ratio/‰ | |
| --- | --- | --- | --- |
|  |  | C | N |
| HH | Cocoon | -27.16±0.23 | 5.54±0.25 |
|  | Degummed cocoon | -27.57±0.29 | 7.62±0.48 |
| HN | Cocoon | -26.91±0.21 | 5.07±0.36 |
|  | Degummed cocoon | -27.62±0.46 | 8.04±0.27 |
| HL | Cocoon | -27.09±0.20 | 5.17±0.45 |
|  | Degummed cocoon | -27.49±0.55 | 5.92±1.32 |
| NH | Cocoon | -27.17±0.18 | 5.37±0.38 |
|  | Degummed cocoon | -28.11±0.48 | 5.64±0.93 |
| NN | Cocoon | -26.96±0.33 | 5.45±0.50 |
|  | Degummed cocoon | -27.43±0.62 | 6.43±1.72 |
| NL | Cocoon | -27.20±0.11 | 5.33±0.27 |
|  | Degummed cocoon | -27.41±0.17 | 7.68±0.73 |
| LH | Cocoon | -27.07±0.29 | 4.98±0.42 |
|  | Degummed cocoon | -28.29±0.19 | 6.86±0.45 |
| LN | Cocoon | -27.17±0.24 | 5.31±0.41 |
|  | Degummed cocoon | -27.81±0.35 | 7.94±0.78 |
| LL | Cocoon | -27.11±0.10 | 5.01±0.32 |
|  | Degummed cocoon | -28.63±0.04 | 7.07±0.68 |


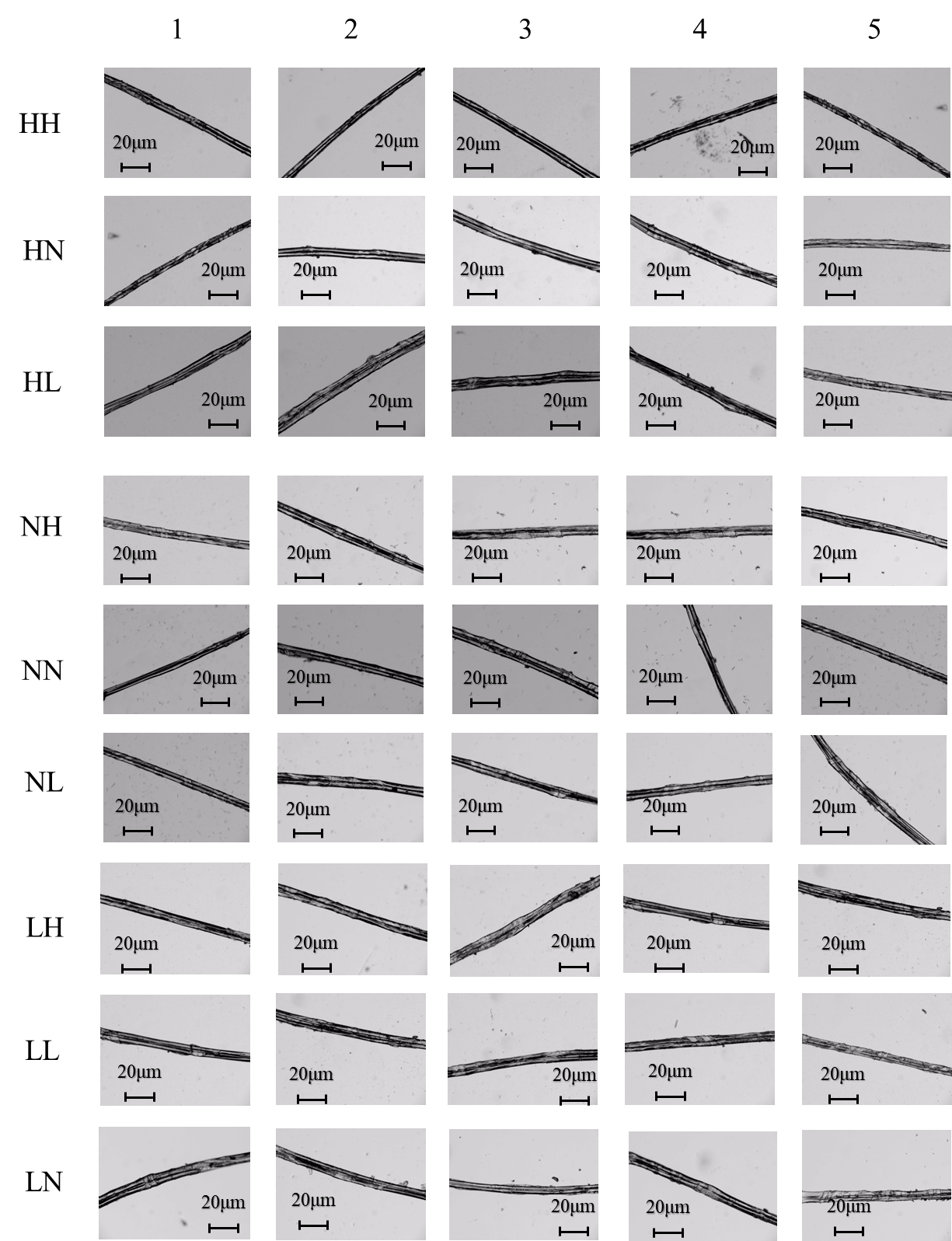


**Figure S1** The optical image of each silk segment from different temperature and humidity conditions.
